# Patient-specific 3D-printed helmet for post-craniectomy defect – a case report

**DOI:** 10.1186/s41205-022-00131-1

**Published:** 2022-01-28

**Authors:** Sherby Suet-Ying Pang, Evan Fang, Kam Wai Chen, Matthew Leung, Velda Ling-Yu Chow, Christian Fang

**Affiliations:** 1 Division of Plastic and Reconstructive Surgery, Department of Surgery, The University of Hong Kong, Hong Kong, China; 2 Department of Orthopaedics and Traumatology, The University of Hong Kong, Hong Kong, China; 3 Prosthetics and Orthotics Department, Queen Mary Hospital, Hong Kong, China

**Keywords:** 3D printing, Skull defect, Protective helmet, Decompressive craniectomy

## Abstract

**Background:**

Patients who undergo decompressive craniectomy (DC) are often fitted with a helmet that protects the craniectomy site from injury during rehabilitation. However, conventional “one-size-fits-all” helmets may not be feasible for certain craniectomy defects. We describe the production and use of a custom 3D-printed helmet for a DC patient where a conventional helmet was not feasible due to the craniectomy defect configuration.

**Case presentation:**

A 65-year-old male with ethmoid sinonasal carcinoma underwent cranionasal resection and DC with free vastus lateralis flap reconstruction to treat cerebrospinal fluid leakage. He required an external helmet to protect the craniectomy site, however, the rim of a conventional helmet compressed the craniectomy site, and the straps compressed the vascular pedicle of the muscle flap. Computed topography (CT) scans of the patient’s cranium were imported into 3D modelling software and used to fabricate a patient-specific, strapless helmet using fused deposition modelling (FDM). The final helmet fit the patient perfectly and circumvented the compression issues, while also providing better cosmesis than the conventional helmet. Four months postoperatively, the helmet remains intact and in use.

**Conclusions:**

3D printing can be used to produce low-volume, patient-specific external devices for rehabilitation where standardized adjuncts are not optimal. Once initial start-up costs and training are overcome, these devices can be produced by surgeons themselves to meet a wide range of clinical needs.

## Background

Decompressive craniectomy (DC) is a neurosurgical procedure used to treat life-threatening elevations in intracranial pressure caused by cerebral edema. Removal of the cranial segment leaves the craniectomy site unprotected during the recovery period. Patients are left at risk for severe brain injury in the event of a traumatic impact to the craniectomy site, with death having been reported as a result [1, 2]. Protective helmets are commonly prescribed to patients who have undergone DC to prevent injury to the craniectomy site during the postoperative period. Conventional helmets are fixed to the head with straps that cross over the chin to prevent slipping and rotation. However, the “one-size-fits-all” design is not always feasible. The use of patient-specific 3D-printed helmets has been reported in the literature for the purpose of improving cosmesis and patient satisfaction [3], but their applicability extends beyond aesthetics. Here, we describe the production and use of a custom 3D-printed helmet for a DC patient where use of a conventional helmet was not feasible.

## Case presentation

A 65-year-old Asian male presented to our centre with a history of lung cancer with brain metastasis. He was treated with radiotherapy in 2002, after which he developed sinonasal carcinoma. He underwent cranionasal resection and craniectomy in February 2020 by an ear, nose and throat surgeon and neurosurgeon following neoadjuvant chemoradiation. Recovery was complicated by cerebrospinal fluid (CSF) leakage from the nose. An attempt was made to repair the defect with temporalis fascia and lumbar drainage of the CSF, however, the leakage persisted. A second repair attempt was made in June 2020 using a fascial graft and a free vastus lateralis muscle flap. Because the superficial temporal vessels had been sacrificed in the previous surgery, the pedicle vessels had to be grafted and anastomosed to vessels in the neck. A 21 cm radial artery graft and a long saphenous vein graft were harvested and anastomosed in end-to-end fashion using interrupted nylon suture. The pedicle artery was anastomosed to the radial artery graft, then to the right facial artery. The pedicle vein was anastomosed to the long saphenous vein graft, then to the internal jugular vein. The pedicle vessel ran through a subcutaneous tunnel along the patient’s right temple and preauricular area. The frontal bone was not returned after craniectomy to avoid strangulating the muscle flap. The patient recovered uneventfully afterwards.

**Fig. 1:**
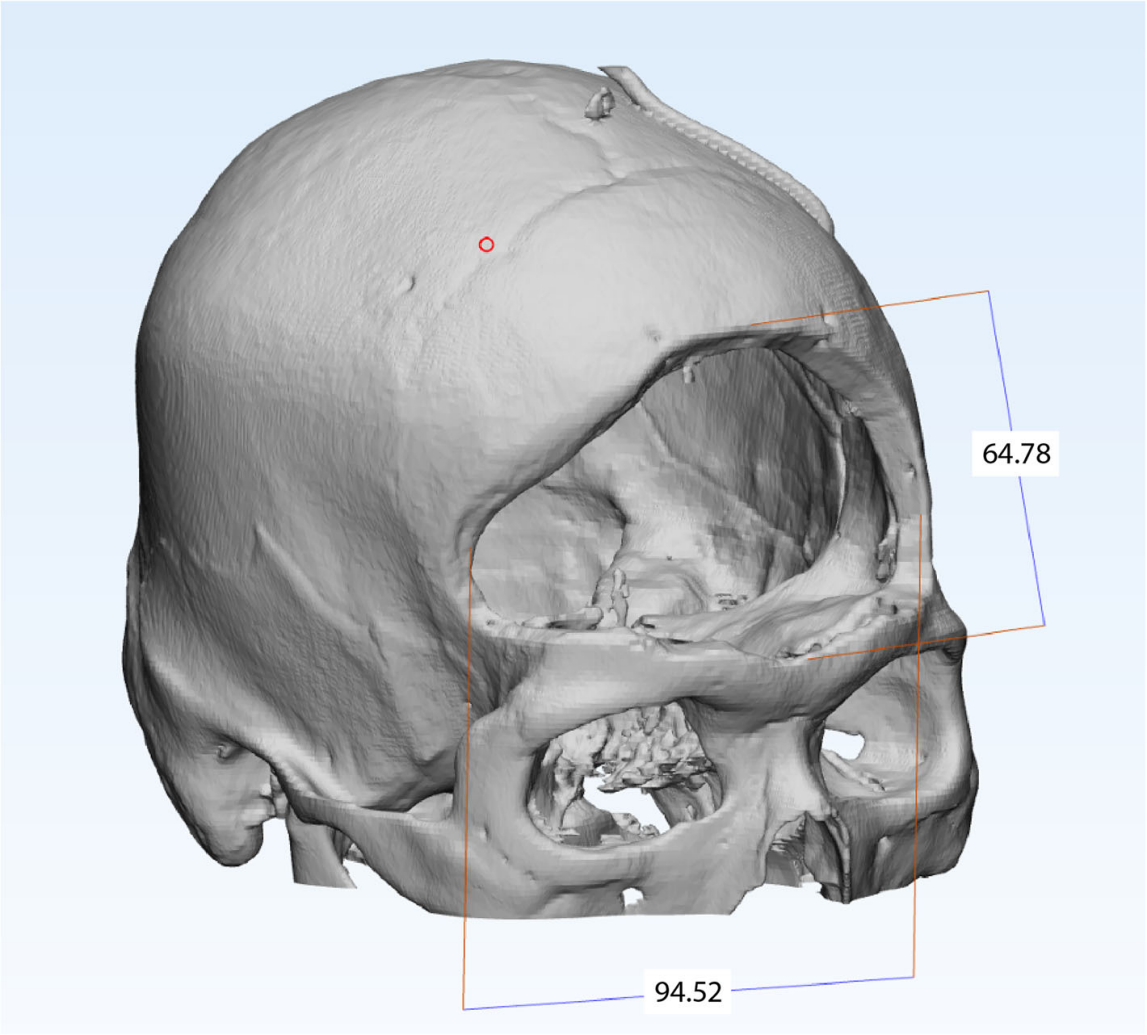
3D rendering of the patient’s skull showing dimensions of the craniectomy defect in millimetres

**Fig. 2:**
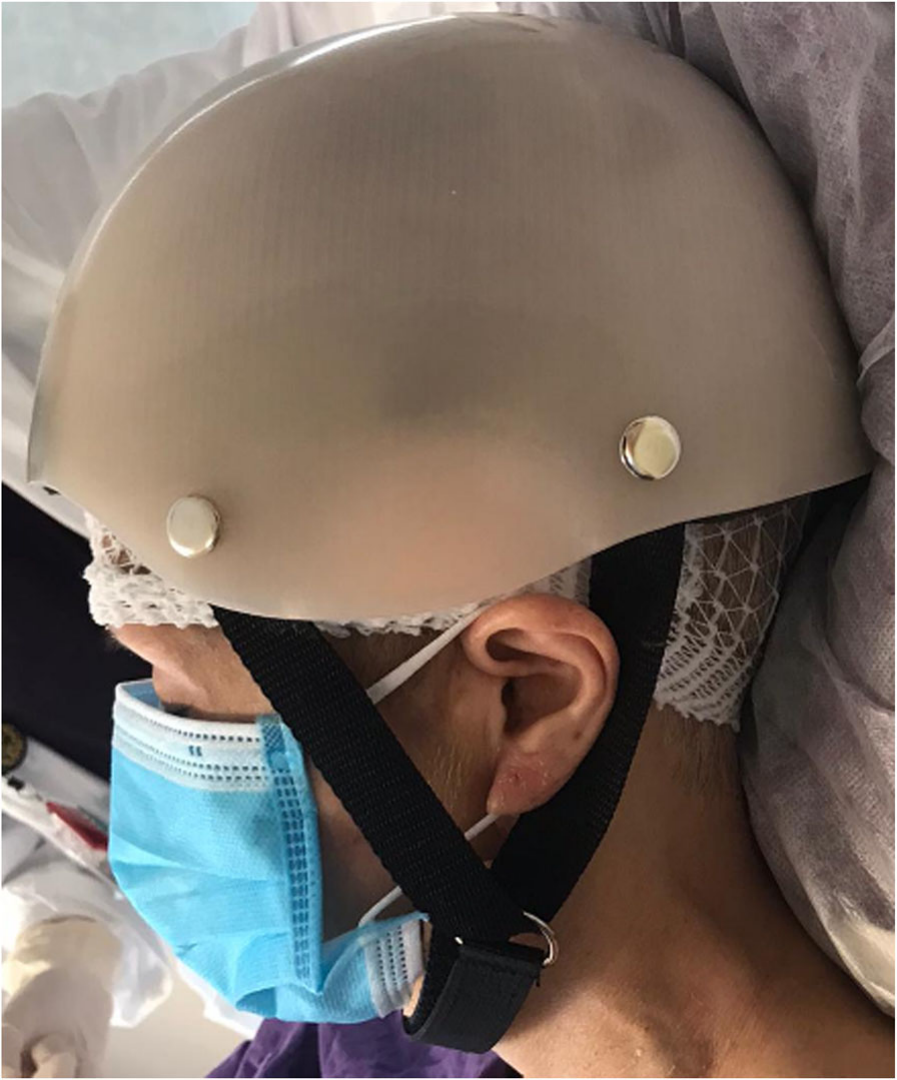
The anterior border of a conventional helmet rests on the craniectomy site and its straps violate the graft pedicle

The present case posed several unique challenges: The resultant frontal skull defect from the supraorbital rim to the vertex measured 64 mm vertically and 95 mm across (Fig. 1). An external helmet was necessary to prevent injury to this region of the brain during rehabilitation. However, our conventional ready-made helmets were deemed unsuitable for two reasons: First, the anterior edge of the conventional helmet landed 1 cm above the eyebrow line and would have violated the craniectomy defect (Fig. 2); second, the conventional helmet was fixed by straps across the temple and submental regions bilaterally, which would have compressed the vein grafts and neck vessel anastomoses. A custom helmet was therefore required, however the potential for compression of these structures to result in complications precluded the possibility of manually casting the patient’s head. Furthermore, the patient was under sedation in his hospital bed, and therefore unable to maintain his posture for 3D scanning. To circumvent these issues, a perfectly fitting, strapless 3D-printed helmet was designed based on pre-existing CT scans of the patient’s skull.

**Fig. 3:**
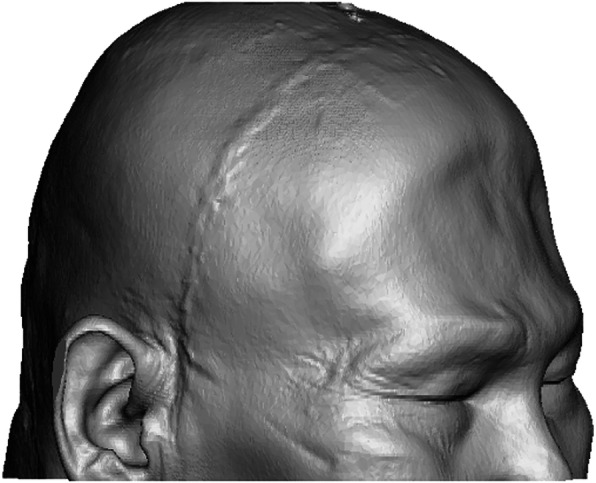
An accurate and detailed 3D skin surface rendering of the patient’s head, based on CT data

**Fig. 4:**
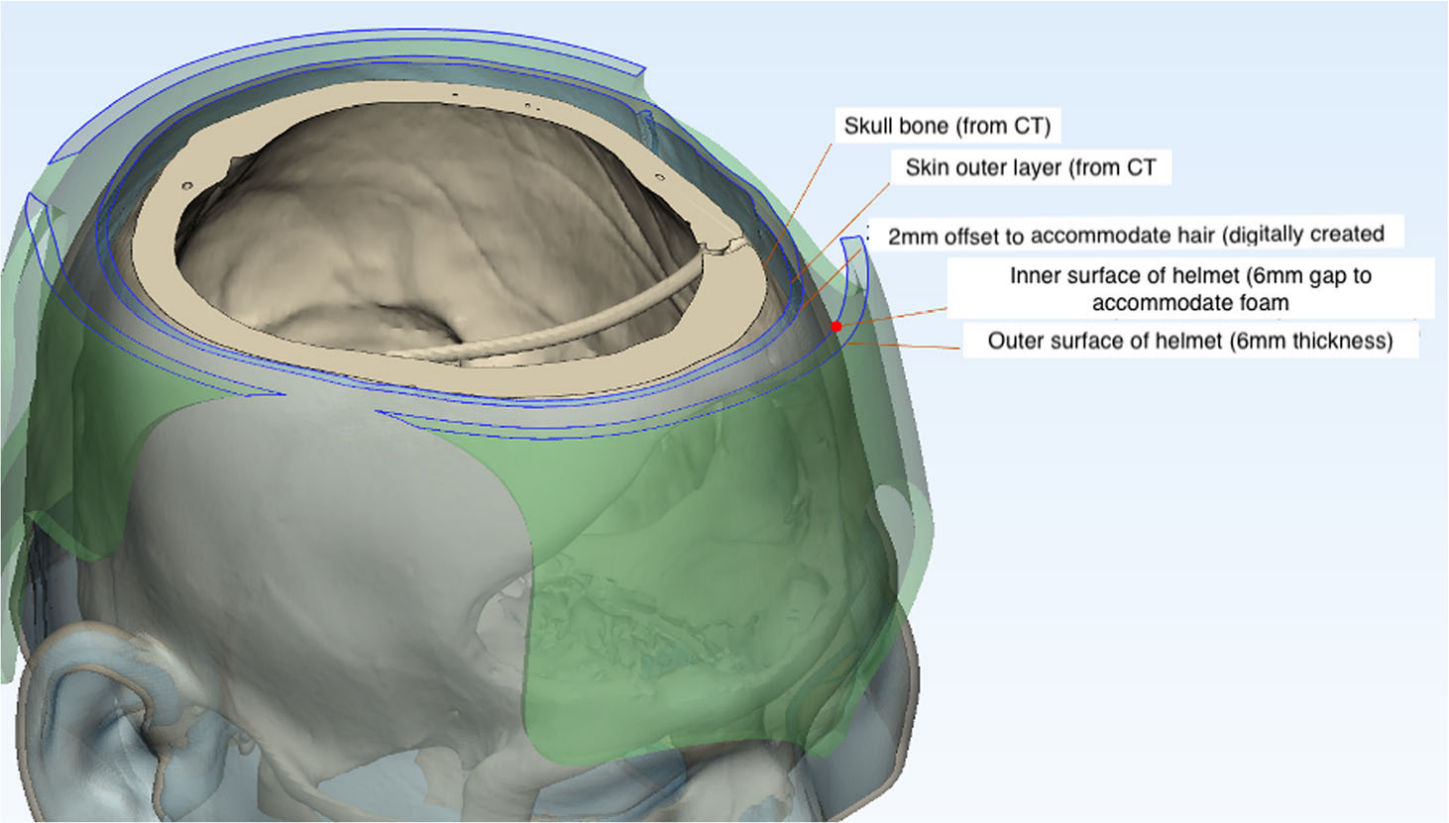
Layers of the head and helmet models in the 3D design process

Computed tomography (CT) scans of the head were obtained in Digital Imaging and Communications in Medicine (DICOM) format with 1 mm slice thickness and a 512 × 512 region of interest (ROI) resolution, corresponding to a voxel size of 0.410 × 0.410 × 1 mm. The images were imported into Mimics v21 software (Materialise NV, Leuven, Belgium) and segmented using the low-noise soft tissue convolution kernel and Hounsfield unit thresholding (Fig. 3). These CT scans were obtained as part of the standard postoperative follow-up for his craniectomy surgery and not specifically for helmet production. A two-layered soft tissue and bony 3D model was created in Standard Triangle Language (STL) format and refined using Meshmixer v3.5 software (Autodesk, San Rafael, CA, USA) and 3-matic v13 software (Materialise). Mesh reduction (decimation), smoothing, and defect filling techniques were applied to the 3D head model to reduce its triangular complexity. A smoothed layer with a 2 mm offset from the skin surface was created to accommodate the patient’s hair (Fig. 4). The model was further smoothed at the craniectomy site to prevent impingement by the helmet and abrasion at the edge of the site.

**Fig. 5:**
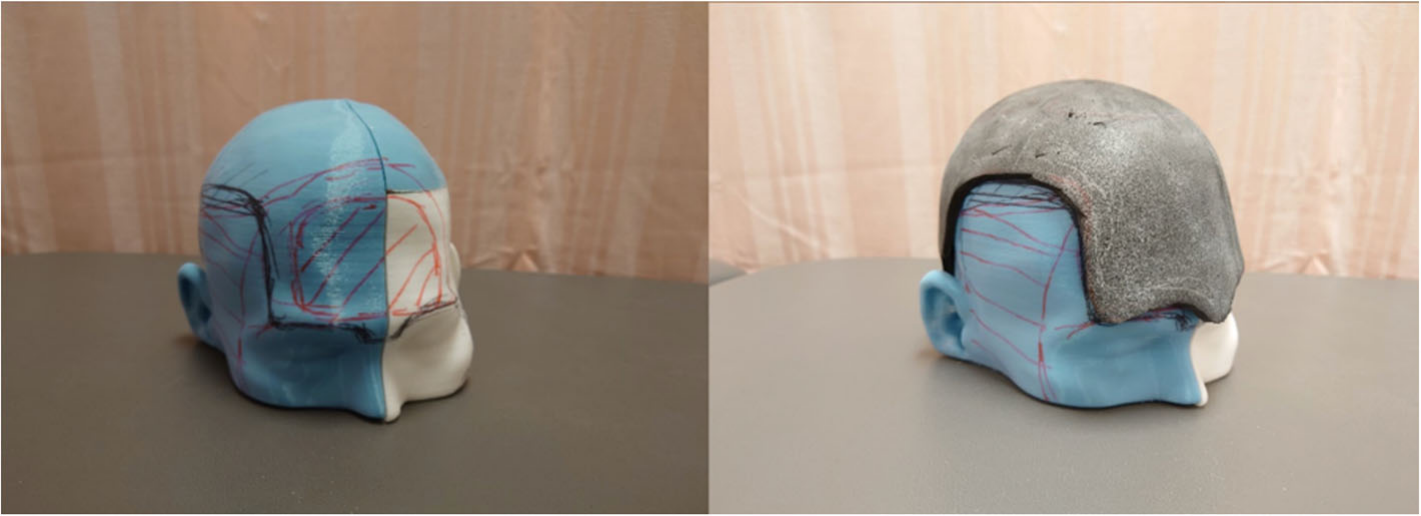
(a) The 3D printed head model, with pressure-sensitive areas marked in red. The helmet border is marked in black (b) The head model fitted with 6 mm of Plastazote® foam

**Fig. 6:**
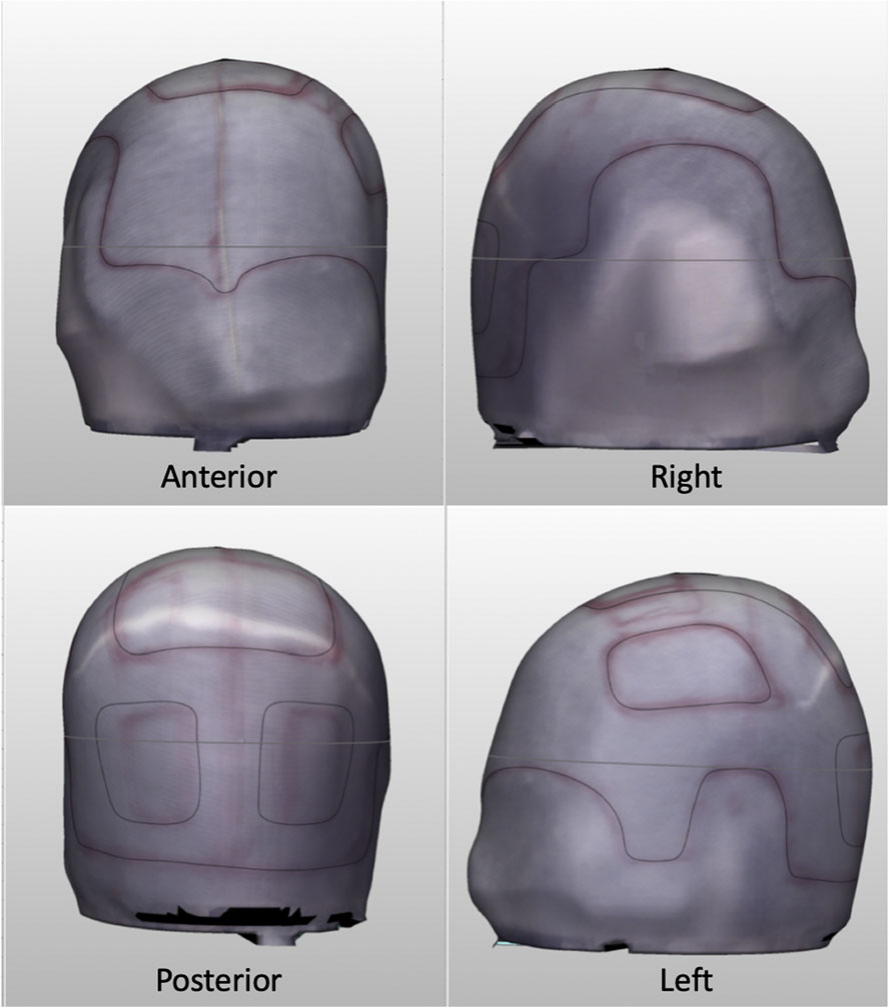
3D-scanned head model with helmet border indicated in red

**Fig. 7:**
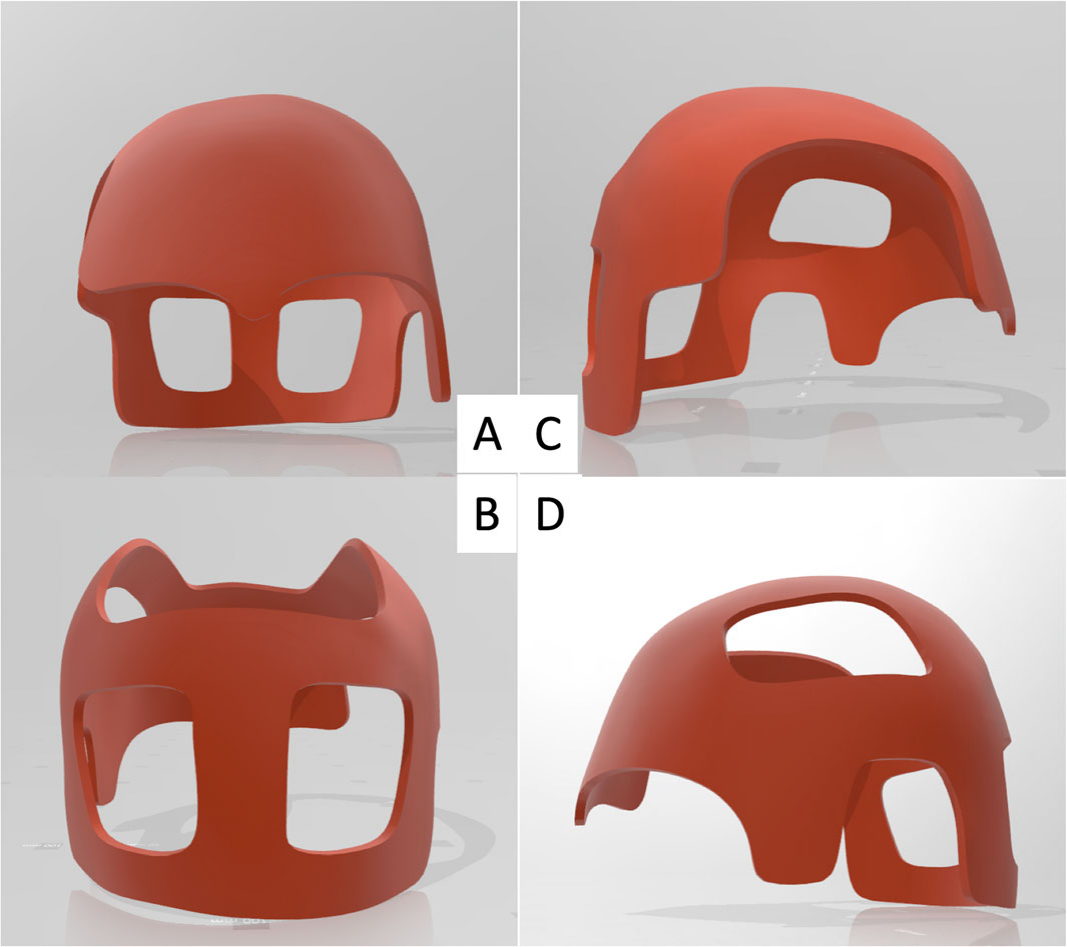
3D helmet prototype: (a) anterior view (b) posterior view (c) right side view (d) left side view

The 3D model of the patient’s head was printed using a desktop 3D printer (DITTO Pro, Tinkerine, Delta, BC, Canada) with non-medical grade polylactide so that fine adjustments could be made to the helmet and foam liner without having to disturb the patient, who was bedridden and uncooperative (Fig. 5a). The helmet was designed to rest on the supraorbital bar and wrap around the vertex and occipital area to avoid compressing the craniectomy defect. A 6 mm layer of Plastazote® foam was fitted to the model and shaped such that the pressure-sensitive areas were avoided (Fig. 5b). A window was created over the right temple and facial area in order to avoid compression of the free vastus lateralis muscle flap pedicle. The foam-fitted head model was scanned using a Spectra 3D scanner (Vorum, Vancouver, Canada). The scanned model was imported to Canfit computer-aided design (CAD) software (Vorum), which was used to define the borders of the helmet and produce a 6 mm-thick prototype helmet model (Figs. 6 and 7).

**Fig. 8:**
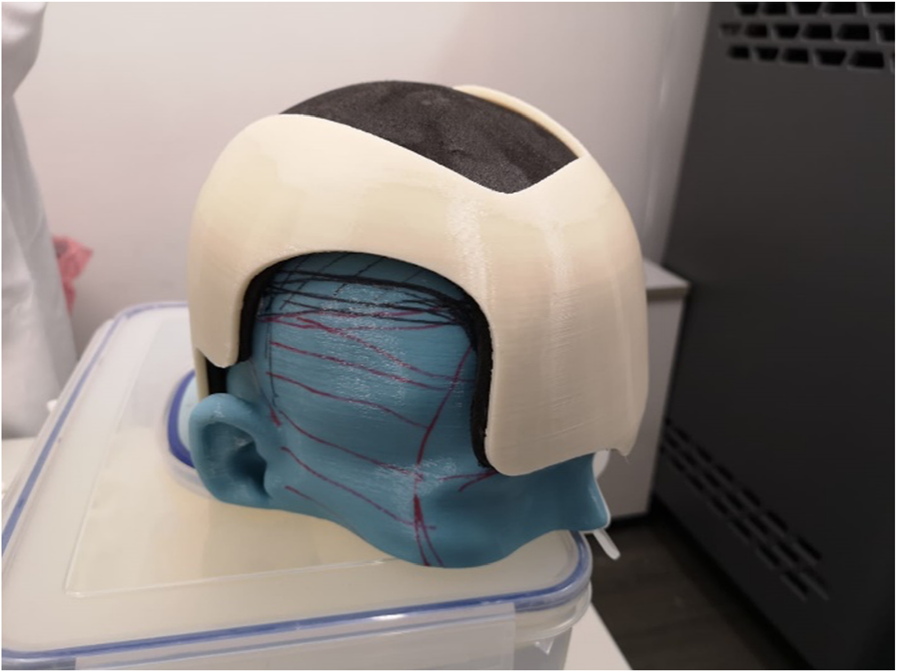
The 3D-printed helmet, with foam insert, fitted to the 3D-printed head model

The finalized helmet model was then 3D printed using medically designated (ISO 10993 and USP Class VI biocompatibility certified) acrylonitrile butadiene styrene (ABS-M30i, Stratasys, MI, USA) by an industrial-grade fused deposition modelling (FDM) printer (Fortus 450mc, Stratasys). The model was printed with a layer thickness of 0.178 mm with a sparse infill density set at 18% to decrease weight and improve patient comfort. The helmet was lined with the foam insert and tested for fitting on the 3D head model (Fig. 8).

**Fig. 9:**
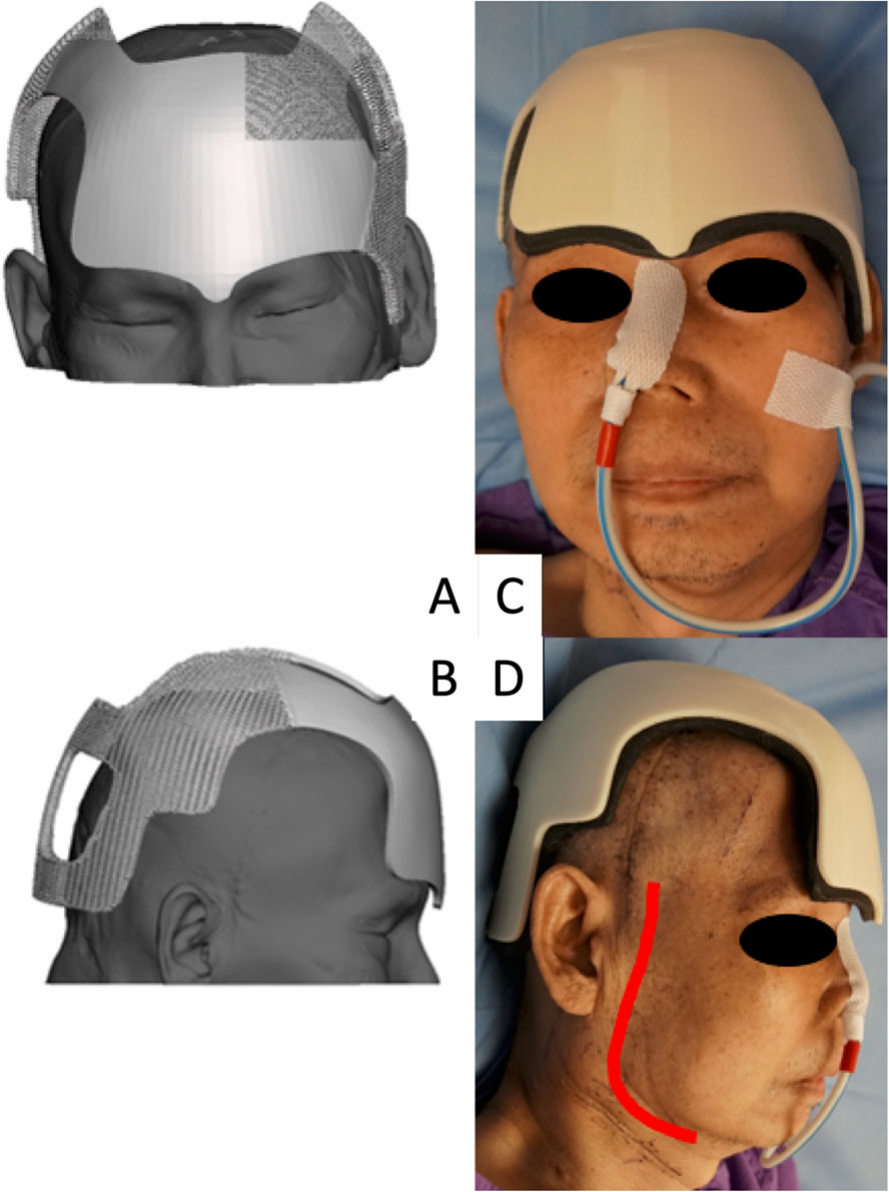
Digital 3D model compared to actual fitting: (a) Anterior view; (b) Side view. Red line indicates position of vascular anastomosis where compression was to be avoided

Upon wearing, the helmet was confirmed to fit properly, without the need for straps to secure it to the patient’s head (Fig. 9). No additional post-processing steps were required. The patient had a prolonged stay in a rehabilitation hospital for physiotherapy. Four months postoperatively, he has undergone three months of daily physiotherapy, including focused lower limb strength training in the form of cycling, and kicking exercises. He can now walk with fair stability using a rollator and the assistance of another person. His Glasgow Coma Scale (GCS) score is E4V2M6 and he remains dependent on others for self-care and activities of daily living. The helmet has proven sufficiently durable and remains wholly intact and in use at the time of writing.

## Discussion

Patient-specific helmets have been in use as early as 1979, when they were first designed to treat plagiocephaly in infants [4]. These early helmets were fabricated by producing a plaster cast of the infant’s head, which was manually reshaped with clay into a more symmetrical head shape, and then fitted with fiberglass or polypropylene to redirect growth of the infant’s skull. Today, helmet therapy is a standard treatment for plagiocephaly, brachycephaly, and postoperative craniosynostosis. Modern orthotic moulding helmets are created with the support of 3D scanning or low dose CT scans and may be produced by healthcare institutions or commercial manufacturers [5]. The emergence of 3D printing and increasing accessibility of computer-aided design (CAD) software has allowed clinicians to produce custom helmets for an increasingly specific and diverse range applications including scalp radiation therapy [6], external fixation of facial fractures [7], orthosis for acrania [8], and improved aesthetics for cranial defects [3]. Our case details the creation of a 3D designed and printed helmet using state-of-the-art methods to overcome a complex clinical scenario. The process and final product allowed us to overcome challenges posed by the unique morphology of the cranial defect and facial grafts, as well as the desire to minimize disturbance of the bed-ridden patient.

In the past, external protective helmets for DC patients have been manufactured in a one-size-fits-all manner with adjustable straps. However, with certain craniectomy defects, these helmets may be unusable. In our case, a conventional helmet would have risked compressing the brain and pedicle vessels and could have led to adverse clinical outcomes. With the use of 3D design, we were able to able to produce a helmet that eliminated contact points over the craniectomy defect. Because the helmet fit the patient’s head perfectly, the need for straps over the blood vessel grafts was eliminated. Patient comfort was also enhanced due to the better fit and lighter weight as compared to conventional helmets. Apart from functional benefit, the 3D-printed helmet also provided better cosmesis than the conventional helmet.

The traditional workflow involves 3D scanning of patient anatomy followed by printing and delivery of the device to verify fit. Additional adjustments are then made based on the results of the fitting, and a subsequent iteration of the device is printed. By printing a model of the patient’s anatomy from previously obtained CT scan, we were able to verify fit and make adjustments without needing to disturb the patient or deliver the prototypical device. This method may be useful for patients who are incapacitated or may otherwise have difficulty in cooperating in the design process.

For commercially sold products, regional jurisdictions in medical device regulations must be considered. As it pertains to the United States Food and Drug Administration (FDA), registration, clearance and premarket approval can be a challenging and time-consuming process. However, under a provision in the Safe Medical Devices Act of 1990, the Humanitarian Device Exemption program permits conditional exemption from certain effectiveness requirements for custom devices intended to benefit patients with conditions appearing in fewer than 8000 people per year in the US [9]. Such conditions do not permit the accumulation of enough clinical evidence to meet FDA standards for safety and effectiveness, and devices intended to treat them are therefore exempted for humanitarian reasons.

The major barriers to implementation of a 3D printing at an institutional level include initial start-up costs and technical training. Our centre uses an industrial grade 3D printer and scanner with costs in excess of USD 200,000. While this represents a substantial initial expense, certain economies of scale such as inter-departmental or inter-institutional utilization and cost sharing can make such an investment more feasible. Our printer provides service to our entire academic institution as well as several smaller centres, and is used extensively by the orthopaedics, craniomaxillofacial and plastics, prosthetics and orthotics, and cardiology departments. In the absence of an in-house printer, printing can also be outsourced to commercial manufacturers once the clinicians have produced the appropriate 3D models. While our team incorporates professional grade software into the design process, our device could have been completed using open source software alone.

**Fig. 10:**
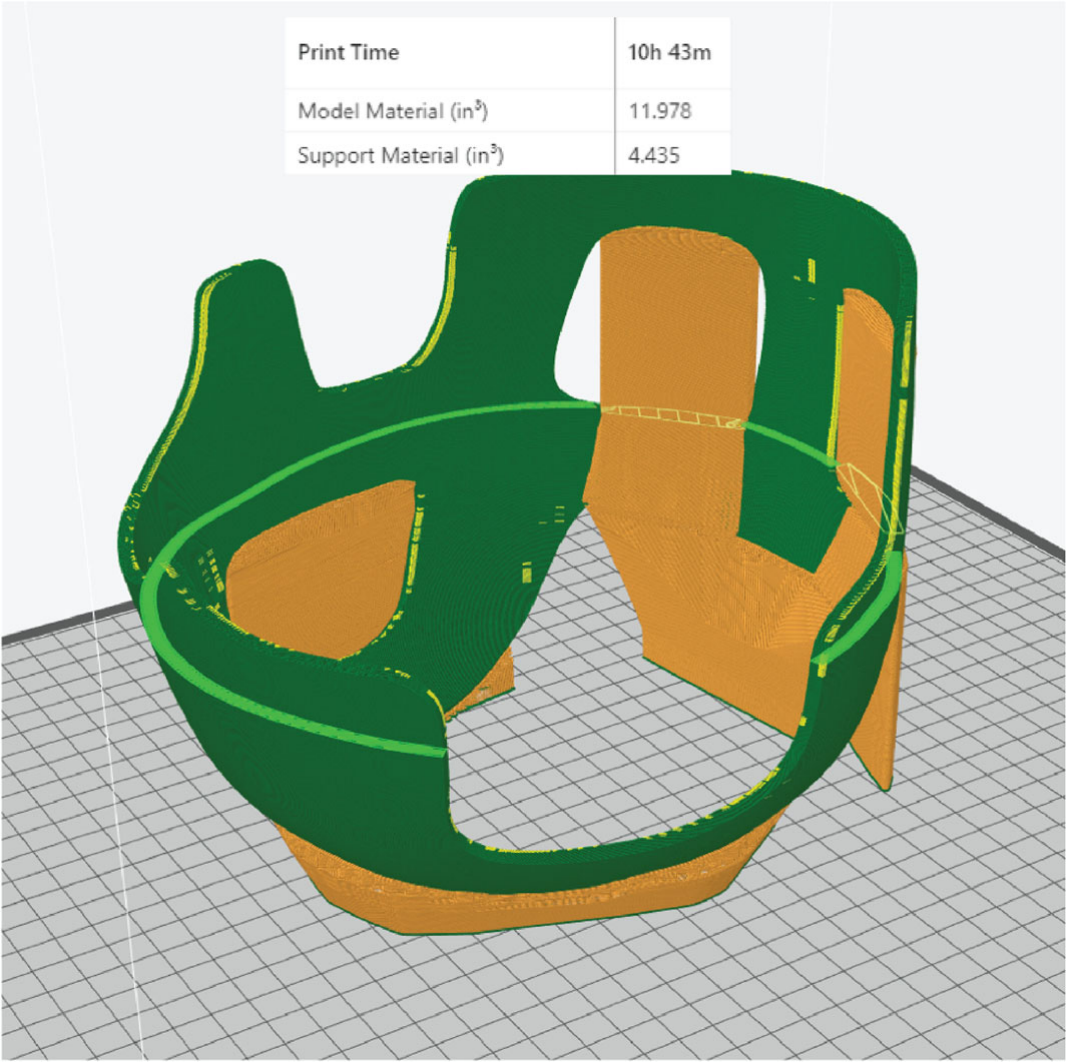
Final 3D helmet model with printing specifications. The central cutaway effectively reduced weight and material use. The final helmet weighed 204 g

The initial time to achieve technical proficiency will vary by clinician, however, once this is overcome, the competent clinician designer can be a valuable interdepartmental resource. Overall, our centre processes around patient-specific devices per year. With initial costs and training barriers overcome, the unit cost of our helmet was around USD 70, design time was approximately 3 h, and machine time was 24 h (13 h for the head model, 11 h for the helmet) (Fig. 10). Only one iteration of the helmet was produced, and the production cycle was completed in under one week by a surgeon designer.

Limitations of our device include that the durability of the material for use as an external device has not been extensively validated by prosthetics and orthotics studies. Furthermore, the helmet offset, and foam thickness measurements were based on experience and remain to be optimized and validated scientifically.

To conclude, our case demonstrates that 3D-printed, low-volume, patient-specific external devices have unique applications in rehabilitation. When initial start-up costs and training are overcome, 3D printing is a diverse technology can be implemented by surgeons themselves to manage a wide range of challenging clinical scenarios.

## Data Availability

Data sharing is not applicable to this article as no datasets were generated or analysed.

## Abbreviations

3D: 3-dimensional
ABS: Acrylonitrile butadiene styrene
CAD: Computer-aided design
CSF: Cerebrospinal fluid
CT: Computed tomography
DC: Decompressive craniectomy
DICOM: Digital imaging and communications in medicine
FDA: Food and Drug Administration
FDM: Fused deposition modeling
GCS: Glasgow Coma Scale
ISO: International Organization for Standardization
ROI: Region of interest
STL: Standard triangle language
USP: United States Pharmacopeia
